# Dental Extractions under General Anesthesia: New Insights from Process Mining

**DOI:** 10.1177/23800844221088833

**Published:** 2022-04-11

**Authors:** F. Fox, H. Whelton, O.A. Johnson, V.R. Aggarwal

**Affiliations:** 1 School of Dentistry, University of Leeds, Leeds, UK; 2 College of Medicine and Health, University College Cork, Cork, Ireland; 3 School of Computing, University of Leeds, Leeds, UK

**Keywords:** dental public health, dental general anesthetic, electronic health records, dental informatics, evidence-based dentistry, patient outcomes

## Abstract

**Introduction:**

Tooth extraction under general anesthetic (GA) is a global health problem. It is expensive, high risk, and resource intensive, and its prevalence and burden should be reduced where possible. Recent innovation in data analysis techniques now makes it possible to assess the impact of GA policy decisions on public health outcomes. This article describes results from one such technique called process mining, which was applied to dental electronic health record (EHR) data. Treatment pathways preceding extractions under general anesthetic were mined to yield useful insights into waiting times, number of dental visits, treatments, and prescribing behaviors associated with this undesirable outcome.

**Method:**

Anonymized data were extracted from a dental EHR covering a population of 231,760 patients aged 0 to 16 y, treated in the Irish public health care system between 2000 and 2014. The data were profiled, assessed for quality, and preprocessed in preparation for analysis. Existing process mining methods were adapted to execute process mining in the context of assessing dental EHR data.

**Results:**

Process models of dental treatment preceding extractions under general anesthetic were generated from the EHR data using process mining tools. A total of 5,563 patients who had 26,115 GA were identified. Of these, 9% received a tooth dressing before extraction with an average lag time of 6 mo between dressing and extraction. In total, 11,867 emergency appointments were attended by the cohort with 2,668 X-rays, 4,370 prescriptions, and over 800 restorations and other treatments carried out prior to tooth extraction.

**Discussion and Conclusions:**

Process models generated useful insights, identifying metrics and issues around extractions under general anesthetic and revealing the complexity of dental treatment pathways. The pathways showed high levels of emergency appointments, prescriptions, and additional tooth restorations ultimately unsuccessful in preventing extractions. Supporting earlier publications, the study suggested earlier screening, preventive initiatives, guideline development, and alternative treatments deserve consideration.

**Knowledge Transfer Statement:**

This study generates insights into tooth extractions under general anesthetic using process mining technologies and methods, revealing levels of extraction and associated high levels of prescriptions, emergency appointments, and restorative treatments. These insights can inform dental planners assessing policy decisions for tooth extractions under general anesthetic. The methods used can be combined with costs and patient outcomes to contribute to more effective decision-making.

## Introduction

Tooth extraction under general anesthetic (GA) is a global health problem. It is both expensive and resource intensive and among the most common reasons for hospital admission in infants and children (World Health Organization 2019). Published figures highlight the prevalence and burden of GA for children on health services. In 2015–2016, approximately 43,700 children were admitted to the hospital in England for the treatment of dental caries, costing £30 million (Knapp et al. 2017). In 2018–2019, there were 44,685 surgical procedures removing more than 1 tooth in those aged 18 and under, the majority driven by tooth decay, costing £41.5 million (The Guardian 2020). The numbers in Ireland are less clear, with the Irish Dental Association (2015) asserting that 10,000 children under the age of 15 were admitted to hospital for GA each year and reports from the Department of Health suggesting a figure of 3,600 annually (RTE 2015). Proportionally, this would be in line with England. GA numbers are also increasing in Australia (Rogers et al. 2018). In addition, potentially avoidable antibiotic prescriptions inadvertently contribute to the global threat of antimicrobial resistance (O’Neill 2014; Viens and Littmann 2015). Further burdens for health services arise through the provision of interim interventions, including temporary dressings and restorations.

In children, it is a traumatic, high-risk procedure and is often preventable (Knapp et al. 2017). It commonly has postoperative symptoms such as pain, agitation, need for analgesics (Needleman et al. 2008), perioperative behavior disturbance (Beringer et al. 2014), and carries risks of morbidity and occasionally mortality. Long waiting times for GA are particularly problematic as children may experience multiple episodes of pain during this time and regular consultations with the primary care dental practitioner are likely necessary (North et al. 2007).

Why are the numbers of GA increasing in the face of falling disease levels? Pinpointing the reasons is problematic and may be due to a variety of reasons, including service availability, dentists’ skill and confidence (Goodwin et al. 2015), or patient/practitioner convenience (Rogers et al. 2018). These numbers could possibly be reduced if children were seen earlier and more frequently by dental professionals for prevention and early intervention (World Health Organization 2019). Use of alternatives to GA, the skill mix of the workforce, and increasing oral health literacy have been identified as factors likely to decrease GA prevalence (Rogers et al. 2018). Greater understanding of the treatment pathways leading up to and including GA is needed in order to determine appropriate public health strategies. However, even if interventions such as these are implemented, evaluating their effectiveness, impact, and outcome remains problematic. Evaluation of population-based prevention in oral health is particularly difficult, especially measuring success by examining changing patterns of disease. Alternative evaluation methods such as the success of the process and screening program participation have been proposed (Daly et al. 2013).

Applying novel analytics methods to large dental data sets can advance these debates on processes and outcomes. The translation of new knowledge discovered by such methods into evidence-based dental practice has potential to improve public oral health outcomes (Finkelstein et al. 2020). Dental electronic health record (EHR) systems, which are used to manage and record the delivery of dental services, provide a rich source of data to which these methods can be applied. Exploratory analysis of these data can build a foundation for detailed modeling of services and outcomes (O’Neil and Schutt 2014). In particular, process mining (PM), an emerging machine learning technique, may prove effective in generating insights into pathways and treatment processes experienced by patients. PM aims to discover, monitor, and improve processes by producing accurate visualizations and generating actionable insights. It is an efficient method to establish what is happening on the ground without employing the traditional tools of observation and questionnaires (Mans et al. 2015). Such care pathway visualizations are a key step in knowledge discovery and learning health care systems (Finkelstein et al. 2020) and are essential for achieving value and continuous quality improvement in oral health care. They have potential to contribute to the development of outcomes and process of care measures (White et al. 2020).

Oral health has been largely ignored by PM, although it has been applied to industry, business (van der Aalst 2011), and health care (Rojas et al. 2016), including specialist areas such as stroke care (Mans et al. 2008), diabetes (Fernandez-LLatas et al. 2015), and oncology (Kurniati et al. 2016). A notable exception are publications emanating from research investigating the transition to digital dentistry (Mans et al. 2012, 2013; van Genuchten et al. 2014). In contrast to prior work, our research applies PM to large-scale dental EHR data, gaining deeper insight into oral health outcomes, particularly GA. This aim of this study is therefore to explore the true burden of GA on patients and health care services using process mining of dental EHR data, developing fresh insights otherwise difficult to elicit from outcome measures or from key performance indicators alone. The specific objectives are first to determine overall treatment patterns preceding GA and, second, to establish the extent and impact of treatments associated with GA, including volumes of antibiotic prescriptions, fillings, temporary dressings, and emergency attendances.

## Method

### Study Design

This study applies a retrospective cohort study with process mining replacing traditional statistical analysis while conforming to applicable Strengthening the Reporting of Observational Studies in Epidemiology (STROBE) guidelines. The cohort all experienced a GA outcome, and the treatment steps preceding this are analyzed to elicit insights about the process leading to GA.

### Population Research Data and Ethics

The research data are an extract from Bridges, a single-center relational database containing information on 231,760 patients’ dental treatment in the Health Service Executive (South), Ireland. It contains data from school screenings and treatments between 2000 and 2014, including attendance records, demographic data, medical history, clinical charting, notes, treatment plans, and dental health status measures. Extracts from the database have been used by the Irish Health Research Board–funded Fluoride and Caring for Children’s Teeth (FACCT) (James et al. 2018, 2020), a project evaluating the impact of policy changes in 2002 and 2007 on children’s oral health, and Mapping the Divide (MTD) (HRA_HSR/2012/25), a project analyzing oral health care services and oral disease levels in children.

The data used formed the basis of the first author’s PhD dissertation (Fox 2019) encompassing exploratory data analysis, data quality assessment, hypotheses generation, and the evaluation of PM’s capacity to extract novel insights from dental EHR data with ethical clearance from University College Cork, reference OHSRC00516, and agreement from the Primary Care Research Committee.

### Data Analysis Steps

Existing PM methods, some addressing the complexities of health care processes, were reviewed and none had been explicitly used in prior dental PM publications (Fox 2019). In order to conduct PM in a dental context, new steps were added to the standard PM^2^ method introduced by van Eck et al. (2015). The final research methodology consisted of 13 steps (Fox 2019). Steps 1 through 8 were general preparatory steps: 1) planning the research, 2) assessing available data, 3) getting appropriate research permissions, 4) preparing and documenting the research environment, 5) extracting data, 6) preprocessing data, 7) assessing data quality, and 8) creating a data description and profile. Steps 9 and 10 defined the specific research objectives and prepared the specific data. In step 11, PM was applied to the data to discover the treatment processes experienced by the GA cohort. Step 12 is the evaluation and discussion of the results, although formal model quality metrics are not generated. Step 13, process improvement and support, is not executed in this research. The results from steps 11 and 12 are the main focus of this article.

### Algorithm and Technology Choices

The priority in selecting the technology was that the resulting models be recognizable and comprehensible to dental experts and that they generate actionable insights. Multiple tests were executed with Disco (version 2.2.1; Fluxicon) and ProM (version 6.6, Revision 28643) on the most commonly used PM algorithms in health care (i.e., Fuzzy Miner, Heuristic Miner, and trace clustering) (Rojas et al. 2016). It was not the intention of this research to formally analyze process models quality metrics, and this encouraged consideration of informal models produced by the Fuzzy Miner and the Heuristic Miner algorithm.

Recognizability and comprehensibility were assessed using a convenience sample of dentists and process miners. Discovered process models were presented at local and international research conferences where they were subjected to scrutiny and debate. This feedback led to some adjustments in the presentation of the models to focus on core issues.

Selecting software technology came down to a choice between ProM and Disco. ProM had become the de facto standard for PM in research. Disco is a commercial product. When comparing use of the Fuzzy Miner, Disco had distinct advantages leading to its selection. It exclusively uses the Fuzzy Miner and delivers a less cluttered and more efficient user interface and functionality while satisfying recognizability and comprehensibility requirements.

## Results

### Summary

Summary analysis of the data set showed 231,760 children had been registered in the EHR since 2000. Of these, 5,563 had a total of 26,115 extractions under GA (January 2004 to December 2013) with extractions peaking at ages 5 and 6. In total, 827 of the patients had a single tooth extracted and 4,736 had multiple extractions.

Comprehensible process models were generated from the EHR data set determining overall treatment patterns preceding GA. Filtering out of unusual activities and pathways was necessary to achieve this. The process models showed 2,165 (9%) of the teeth extracted had received a tooth dressing before GA. The mean time between dressing and extraction was 29.3 wk. The models showed notable levels of restorative and other treatments preceding GA. Similar treatment pathways existed for permanent and deciduous teeth. In addition, 11,867 emergency appointments, 4,370 prescriptions, 2,668 X-rays, and 4,942 referrals for GA and oral surgery were recorded for the cohort showing other service impacts of GA.

### Planning and Data Preprocessing

The data were stored within the secure data virtual research environment of Leeds Institute for Data Analytics, encrypted and accessible only to the research team (Fox 2019). Data preprocessing consisted primarily of anonymization (Fox 2019). Data were assessed in the context of the research question, and data of deficient quality were marked and excluded from the analysis where appropriate. The data were evaluated under 4 broad data quality issues existing in event logs: missing, incorrect, imprecise, and irrelevant (Bose et al. 2013; Mans et al. 2015). Not all issues disqualified the data for all questions, and this complexity was managed through development of a data quality management framework (Fox et al. 2018). The data were described and profiled using exploratory data analysis (O’Neil and Schutt 2014).

Data aggregations were created to support the research questions (Fox 2019). Although data were available from 2000, the EHR was not being used consistently and throughout the target area until 2003, so this analysis used data in the 10-y period between January 1, 2004, and December 31, 2014. Detailed data entry protocols and standard operating procedures were being developed in early years, and these data were excluded as a run-in period. Teeth extracted under GA and all prior treatment events experienced by these teeth were exported for analysis and process mining. Key technologies used were Disco, ProM, Python, SQL, and Microsoft SQL Server.

### Number of Children, Demographics, and Waiting Times for GA

**Figure 1.**
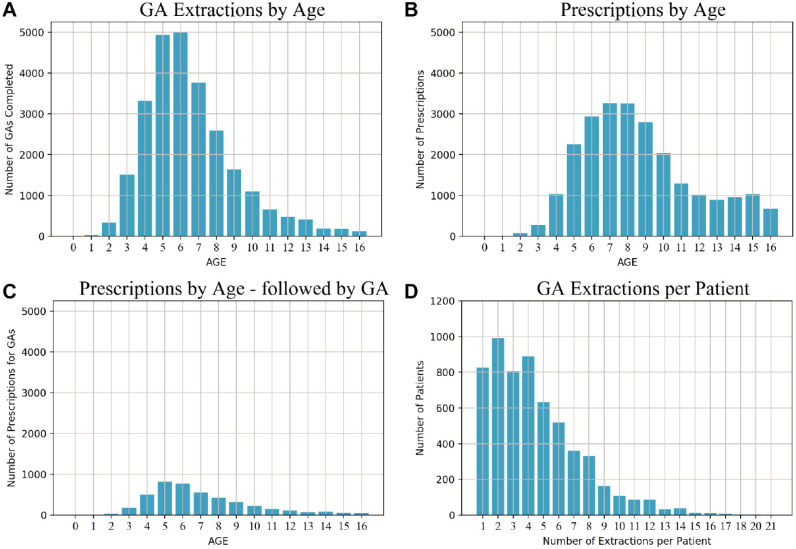
Age at GA and prescriptions profile. (**A**) Number of general anesthetic (GA) extractions by age. (**B**) Number of prescriptions by age. (**C**) Number of prescriptions by age, followed by GA. (**D**) Number of GA extractions per patient.

In total, 231,760 children were registered in the EHR. Analysis showed high levels of preventive treatments (e.g., 326,803 screenings and 694,650 fissure seals) in addition to restorative treatments (e.g., 221,330 amalgams and 123,139 composite fillings). The age profile of 5,563 patients having at least 1 GA is shown in Figure 1A with extractions peaking at ages 5 and 6. The distribution of prescriptions by age, shown in Figure 1B, C, shows prescriptions followed by GA. Figure 1D shows the distribution of numbers of extractions per child. This shows the highest rates of GA occurring around the age children would normally initially engage with the public system for screening and preventive treatment. No earlier intervention was recorded in the EHR for 88% of the teeth extracted, and this aligns with an earlier study in which, of 347 children experiencing dental general anesthesia, 306 had an emergency appointment as their first dental visit (McAuliffe et al. 2017).

**Figure 2.**
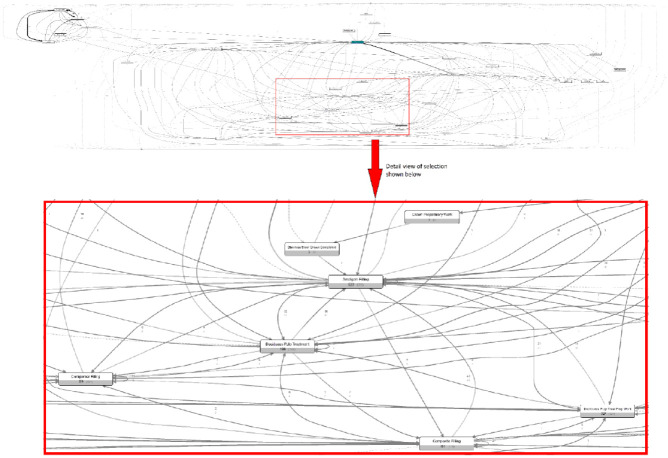
Unfiltered process model demonstrating the complexity of dental treatment processes and the resulting “spaghetti” appearance. Overview of the full pathway model for general anesthetic from the Bridges data extract with a closeup view of paths leading to a composite filling detail in the red box. (Produced using Fluxicon Disco v 1.6.)

**Figure 3.**
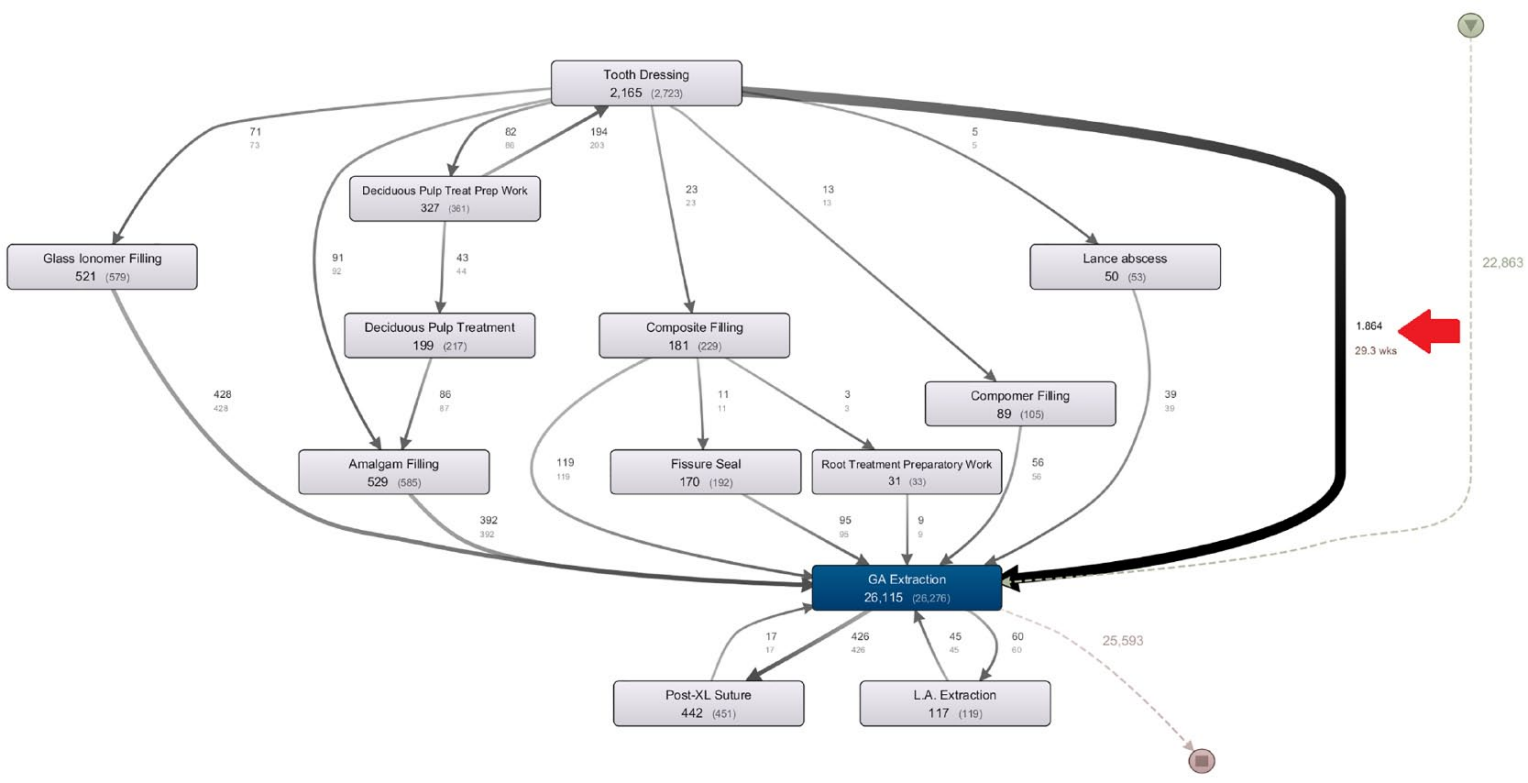
Process mining frequency analysis of tooth extractions under general anesthetic (GA). Temporal sequence for teeth extracted under GA between 2004 and 2014 and all preceding events. (Produced using Fluxicon Disco v 1.6.)

Applying the PM fuzzy algorithm to the complete data set identified 532 different pathway variations experienced by the cohort. Displaying all pathways and activities yielded an incomprehensible “spaghetti” model shown in Figure 2. Interpreting this model is almost impossible due to its overwhelming level of detail, highlighting a common problem when process mining health care data (i.e., the inherent complexity of health care processes with an often unique care pathway experienced by patients) (Mans et al. 2008; Rovani et al. 2015). Model legibility and comprehensibility were attained by omitting less frequently occurring activities and pathways (Fox 2019). The model in Figure 3 represents the most common pathways followed by teeth extracted under GA. The detail level for the following models was set in the Disco user interface (activities = 50%, paths = 25%). It is important to note that this filtering could exclude potentially important pathways, but detailed examination of the spaghetti model did not reveal other interesting variations or expose common treatments not represented in Figure 3. Nevertheless, excising data must be done cautiously because complex or convoluted treatment pathways can be more expensive and therefore of interest.

The discovered process models showed that 26,115 teeth were extracted under GA in this 10-y time period (Fig. 3). Of these teeth, 22,863 had no other tooth-specific intervention recorded in this data. These are represented by the light, dashed pathway directly to “GA Extraction” on the right-hand side of the process model. Over 9% (2,165 instances) of the teeth received a tooth dressing before GA and the average time between a tooth’s first dressing and GA was 29.3 wk, highlighted by the red arrow in Figure 3.

The model gives us information on treatments administered in the time between “Tooth Dressing” and “GA Extraction.” Of the extracted teeth, 529 teeth received an amalgam filling on average 20.3 mo prior to extraction, and 392 of these went directly to GA without any intervening treatment. The remaining 137 took an alternate path to GA not shown. In addition to the amalgam fillings, a further 181 received a composite filling and 89 received a compomer filling. A further 521 are indicated with a glass ionomer filling as a separate treatment item to the “Tooth Dressing.”

The model shows both deciduous and permanent teeth. As expected, most extractions in the model were deciduous teeth (90.3%). Separate modeling of permanent tooth extractions showed similar treatment pathways to deciduous teeth. However, tooth dressings were almost twice as prevalent: 18.67% versus 9.46% of deciduous teeth. Also, 11.6% had fillings prior to extraction versus 2.5% of deciduous teeth. This is not unexpected as children with permanent teeth are older and more likely to have experienced prior oral health care.

### X-Rays and Prescriptions

**Figure 4.**
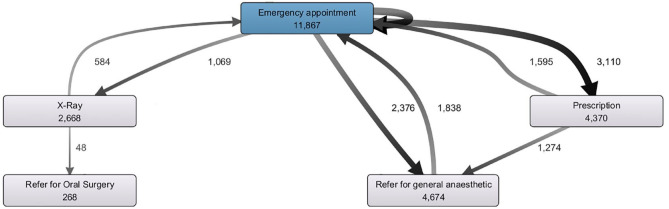
Process model perspective of the general anesthetic patient’s care pathway highlighting treatment events not associated with a specific tooth. (Produced using Fluxicon Disco v 1.6.)

Figure 4 shows an alternative perspective of the GA patient’s care pathway, highlighting events linked to the patient but not associated with a specific tooth. The model shows appointments, prescriptions, and X-rays. Notable events are the 11,867 emergency appointments, 4,370 prescriptions, 4,942 referrals for GA and oral surgery, and 2,668 X-rays.

## Discussion

The models highlight elements of the service deserving the attention of policy makers. First, the models showed that 87.5% of the teeth were extracted without any prior recorded intervention specific to that tooth, suggesting that earlier screening and prevention should be considered, particularly for high caries risk groups. Supporting this, Rogers et al. (2018) found that the principal diagnosis for over 90% of potentially preventable dental hospitalizations was dental caries, where “potentially preventable” meant avoidable if timely and adequate nonhospital care was provided or if the condition could have been prevented in the first place. They proposed that developing guidelines and using alternative approaches would decrease the prevalence of dental general anesthetics. In this regard, the use of a silver diamine fluoride (SDF) protocol has been shown to be effective (Yawary and Hegde 2021). If implemented, the impact of the SDF protocol should also be visible in the models. Development and implementing of guidelines for employing general anesthetic in dental settings would add consistency to the diagnosis process. In addition, 827 of the patients had a single tooth extracted, while 4,736 had multiple teeth extracted, further supporting the incorporating of both pre- and postextraction preventive interventions in the process (Savanheimo and Vehkalahti 2014) with the potential to improve oral health literacy and avoid future extractions (Goodwin et al. 2015).

Second, the presence of restorations in the care pathway preceding extractions raises the policy questions as to whether or not these interventions improve outcomes (Milsom et al. 2002). Also prominent in the models is the high level of use of emergency appointments preceding GA, which is likely an unwelcome and disruptive burden on routine dental services, as are referrals for oral surgery and general anesthetic. In addition, the models highlight the use of potentially avoidable antibiotic prescriptions (North et al. 2007), raising the issue of the impact on antimicrobial resistance.

Finally, the 6-mo time lag between dressing and extraction of teeth for 9% of the teeth raises an issue for dental care providers to explore and seek solutions, as prolonged wait times are known to have negative effects such as pain, antibiotic use, sleepless nights, and missed school time (North et al. 2007; Goodwin et al. 2015). For example, access to general anesthesia facilities was identified as a factor affecting waiting times in Australia (Rogers et al. 2018). The model does not explain this time lag, although it was comparable to an average wait time of 8 mo in an observational study in England (Goodwin et al. 2015), although somewhat longer than a survey study where the best estimates of usual waiting times found most to be shorter than 18 wk (Ní Chaollaí et al. 2010).

### Limitations of the Data and the Models

While there is significant support for the secondary use of EHR data in research (EU 2018; National Institute for Health and Care Excellence 2020), questions remain about the suitability of such data to answer public health policy questions. Difficulties can be attributed to confounders such as social determinants of health (World Health Organization 2020) not always being visible in real-world data. Health is a multifactorial concept, and health care intervention is not the only factor influencing health care outcomes.

Specific to this study, the analysis was limited by the scope of the data that could be extracted from the dental EHR and its quality. The data were anonymized in advance of analysis, inevitably removing some value (e.g., dates of birth and address) that could have facilitated socioeconomic analysis. Furthermore, the data encompass only those who attended the public health dental service from 2000 to 2014. Results are specific to the location and the time. Other patients may have attended private dental services exclusively, and some may have attended both. Also, this analysis did not account for the existence of prior oral examinations or treatments of other teeth. The date of the decision to recommend GA would have given additional valuable information about the time lag before GA, but this was not available. The slight discrepancy between the total number of GA and the sum of the teeth in the paths followed in the model is due to the exclusion of unusual paths and events. It is also notable that a small number of teeth are marked as having had a local anesthetic extraction in addition to the GA, and this is most likely a data recording error where a tooth number was incorrectly identified. Furthermore, the multifaceted nature of EHR data quality is a limitation inherent in secondary use (Fox et al. 2018).

In addition to the above data limitations, the PM fuzzy modeling technique is not deterministic and provides an estimate of likely pathways supported by event statistics. It requires tuning to achieve comprehensible models and requires care in execution and documentation. Although the convenience sample of dentists provided valuable feedback on the recognizability and comprehensibility of the models, an ideal experiment would see a representative sample of dentists presented with process models of various familiar scenarios, and their ability to recognize and comprehend the models accurately and in a timely manner would be recorded, giving a more scientific assessment of the models’ quality characteristics. This approach would merit consideration for future work.

Also notable is that none of the authors was involved in the clinical decisions generating the EHR data, and due to this degree of separation, this study is limited in the strength of the clinical conclusions that can be drawn. However, it could be also argued that this brings a welcome degree of objectivity.

### Advantages of the Process Mining Approach

From our review of the literature, this is the first successful application of PM to data from a public health dental EHR. This research emphasized diligent data preparation, data quality analysis, and their documentation prior to process mining (Fox et al. 2018; Fox 2019). This upfront investment generated confidence in the quality of the data. PM then facilitated rapid analysis of the large dental data set, creating process models and generating insights and initial hypotheses efficiently versus the more traditional survey or observational methods in prior studies (Ní Chaollaí et al. 2010; Goodwin et al. 2015; Rogers et al. 2018). The automated features of the infrastructure and method also allowed repeated experiments with differing age cohorts and selection criteria with minimum effort.

### Future Work

While this study focused on a retrospective analysis of EHR data, PM could be used in practice-based clinical trials using patient randomization showing care pathways leading to treatment outcomes or study intermediate steps leading to outcomes from using various treatments, interventions, materials, or drugs. PM could add further value to public health data analysis by showing the impact on outcomes of adding resources to a service. The approach showed the potential to quickly uncover relationships between steps in the process model and provide hypotheses for further study.

## Conclusions

### Clinical Implications

The analysis in this research reveals a number of areas deserving consideration by policy makers. First, earlier screening and prevention should be considered, particularly for high caries risk groups, as the models showed that most teeth were extracted without any prior recorded intervention specific to that tooth. Also, arising from the prevalence of multiple extractions for the majority of patients, an opportunity exists for both pre- and post-extraction preventive interventions to improve oral health literacy and avoid future extractions. Furthermore, highlighting the presence of restorations in the process preceding extractions raises the question of the value of these interventions. In addition, recent research has highlighted the value of guideline development in this area and the effective use of alternative treatments such as silver diamine fluoride. Finally, the findings that patients experiencing GA also had a large number of emergency appointments and a large volume of potentially avoidable antibiotic prescriptions suggest a need to reduce GA waiting times possibly by increasing service availability and further public health prevention measures.

### Conclusion

This study analyzed tooth extractions under general anesthetic using PM of electronic health records to visualize the care pathways experienced by patients. It revealed the levels of GA and preceding treatments, including associated high levels of prescriptions, emergency appointments, and restorative treatments recorded for the population in the EHR.

Applying PM to dental EHR data showed the potential to facilitate more thorough analysis of the effects of public health interventions by looking behind the outcomes, easily generating concise visualizations of processes in previously unavailable detail.

The results of this study can be used by dental planners and policy makers when assessing decisions and factors affecting tooth extractions under general anesthetic. The approach helps in identifying issues in processes and establishes a basis for assessing management and policy decisions through rapid prototypes and model visualizations. The study suggested that earlier screening, preventive initiatives, guideline development, and alternative treatments deserve consideration.

## Author Contributions

F. Fox, contributed to conception, design, data acquisition, analysis, and interpretation, drafted and critically revised the manuscript; H. Whelton, contributed to conception, design, data acquisition, analysis, and interpretation, critically revised the manuscript; O.A. Johnson, contributed to conception, critically revised the manuscript; V.R. Aggarwal, contributed to conception, data analysis and interpretation, critically revised the manuscript. All authors gave final approval and agree to be accountable for all aspects of the work.
